# The first clinical data of the SAPIEN 3 aortic valve in the treatment of aortic stenosis in China

**DOI:** 10.3389/fcvm.2023.1064255

**Published:** 2023-06-13

**Authors:** Xiaoke Shang, Xiangbin Pan, Gejun Zhang, Zhengming Jiang, Xianbao Liu, Guangyuan Song, Yi Li, Yan Wang, Jianfang Luo, Yida Tang, Yiqiang Yuan, Yongjian Wu, Xiang Ma, Dan Zhu, Yucheng Zhong, Changdong Zhang, Nianguo Dong

**Affiliations:** ^1^ Department of Cardiovascular Surgery, Union Hospital, Tongji Medical College, Huazhong University of Science and Technology, Wuhan, China; ^2^ Department of Cardiovascular Surgery, Fuwai Hospital, CAMS&PUMC, Beijing, China; ^3^ Department of Cardiology, The First Affiliated Hospital of Zhengzhou University, Zhengzhou, China; ^4^ Department of Cardiology, The Second Affiliated Hospital of Zhejiang University School of Medicine, Hangzhou, China; ^5^ Department of Cardiology, Beijing Anzhen Hospital, Capital Medical University, Beijing, China; ^6^ Department of Cardiology, The First Affiliated Hospital, Sun Yat-sun University, Guangzhou, China; ^7^ Department of Cardiology, Xiamen Cardiovascular Hospital, Xiamen University, Xiamen, China; ^8^ Department of Cardiology, Guangdong Provincial People’s Hospital, Guangzhou, China; ^9^ Department of Cardiology, Peking University Third Hospital, Beijing, China; ^10^ Department of Cardiology, Henan Provincial Chest Hospital, Zhengzhou, China; ^11^ Department of Cardiology, Fuwai Hospital, CAMS&PUMC, Beijing, China; ^12^ Department of Cardiology, The First Affiliated Hospital of Xinjiang Medical University, Urumqi, China; ^13^ Department of Cardiovascular Surgery, Shanghai Chest Hospital, Shanghai, China

**Keywords:** transcatheter aortic valve replacement (TAVR), bicuspid aortic valve (BAV), tricuspid aortic valve(TAV), Edwards SAPIEN 3, aortic stenosis (AS)

## Abstract

**Background:**

Data on outcomes following transcatheter aortic valve replacement with SAPIEN 3 in China is limited as it was approved by the National Medical Products since 2020. The present study was designed to collect clinical data on the SAPIEN 3 aortic valve in Chinese patients with bicuspid aortic valve and tricuspid aortic valve stenosis.

**Methods:**

We analyzed the patient characteristics, procedural features and procedural outcomes of the first 438 patients (223 for bicuspid aortic valve and 215 tricuspid aortic valve) from 21 provinces in 74 sites treated with the SAPIEN 3 valve system for transcatheter aortic valve replacement between September 2020 and May 2022.

**Results:**

Procedural mortality was 0.7%. 5 cases during the operation were converted to surgery. Among 438 cases, permanent pacemaker implantation was performed in a total of 12 cases (2.7%). The patient had severe leaflet calcification of the aortic valve, with moderate and severe calcification reaching 39.7% and 35.2% respectively. The size of the implanted valves was predominantly 26 mm and 23 mm, reaching 42.5% and 39.5% respectively. The incidence of moderate or severe perivalvular leak in the postoperative period was 0.5%, with a predominance of 90/10 and 80/20 valve deployment height. There was a significant difference in the deployment height of the valve between bicuspid aortic valve and tricuspid aortic valve, with the bicuspid aortic valve having a more deployment height of 90/10. Annulus size in bicuspid aortic valve group was significantly larger than tricuspid aortic valve group. Valve sizing for oversized, within size, and undersized were different between bicuspid aortic valve and tricuspid aortic valve.

**Conclusions:**

Procedural success rates were high, with similar and good results for bicuspid aortic valve and tricuspid aortic valve, low perivalvular leak for both valve types, and low permanent pacemaker implantation rates for both valve types. Annulus size, valve sizing and coronary artery height were significantly different in the BAV and TAV group.

## Introduction

Aortic stenosis (AS) is one of the most common acquired heart valve disease, with a prevalence of over 2% of patients over 60 years of age, which is generally accompanied by a high mortality rate when it is severe and not treated with aortic valave replacement (1, 2). According to the number of leaflets, AS can be classified as bicuspid stenosis and tricuspid stenosis, of which the bicuspid stenosis is more prevalent (3). Data from 2004 showed that the prevalence of bicuspid aortic stenosis among adolescents was 1%, more than half of the patients require surgical treatment (4). Transcatheter aortic valve replacement (TAVR) has become the well-established treatment for AS (5). The main types of prosthetic arterial valves at present are self-expanding valves (SEV) and balloon-expanding valves (BEV) (6, 7). BEV are the most used in the developed world and have a rich clinical database (8). In Asian countries, the Edwards SAPIEN (Edwards Lifesciences) series of BEV is the most commonly used, followed by SEV. 90.3% and 9.7% of TAVR procedures in Japan use BEV and SEV, respectively. However, the relative proportions of these two most commonly used valves may vary considerably from country to country (9). However, due to the smaller physical size of the Asian population and their smaller valves, there is a relative lack of clinical data on BEV (10). Especially in China, to date, 95% of patients are clinically treated with self-expanding valves, and even the expert consensus on treatment is for SEV (11). SAPIEN 3 Transcatheter Aortic BEV System was approved by the National Medical Products Administration in China just since June 2020 (12). A study at Fu Wai Hospital in China showed a high safety and efficacy profile for BEV (SAPIEN 3) and SEV, with no significant difference, however, the number of BEV cases in this study was small (*n* = 25) (13). Therefore, more robust clinical data on BEV in China is still lacking.

The proportion of bicuspid stenosis in aortic valves is significantly higher in Chinese than Western population, and although BEV solutions for bicuspid aortic valve are now being explored abroad, early clinical trials of BEV excluded bicuspid aortic valve (BAV) stenosis (14). Thus, global clinical data on the treatment of BAV with BEV are still lacking.

Our study collected clinical data from the first 438 Chinese patients with AS, including 223 patients with bicuspid aortic stenosis. This study provides the first clinical data on BEV in China, and we compare outcomes in BAV vs. TAV stenosis, which enriches the global clinical data on the treatment of AS.

## Methods

All consecutive patients implanted with SAPIEN 3 (Edwards Lifesciences, Irvine, CA) after SAPIEN 3 received NMPA approval for the first commercial case in September 2020–May 2022 were counted. This study was approved by the ethics committee of Wuhan union hospital (2020-0569-06).

Inclusion and exclusion criteria mainly referred to the expert consensus on clinical pathway for transcatheter aortic valve replacement in China (11).

### Inclusion criteria

Patient with symptomatic aortic stenosis due to severe native calcific aortic stenosis requiring aortic valve replacement and undergoing TAVR with the Edwards SAPIEN.

### Exclusion criteria

Patients or their guardians do not consent to enquiries or follow-up visits due to medical, social or psychological conditions.

Patients with active SARS-CoV-2 infection [Coronavirus-19 (COVID-19)] or previously diagnosed with COVID-19 with sequelae that could confound endpoint assessments.

Cannot tolerate an anticoagulation/antiplatelet regimen.

Evidence of intracardiac mass, thrombus, vegetation, active infection or endocarditis. Tortuous or calcified vessels that would prevent safe entry of the dilators and sheath.

Participating in a drug or device study that has not reached its primary endpoint.

Patient selection for TAVR has been described previously (15). Patient with severe symptomatic aortic stenosis (an aortic-valve area ≤1 cm^2^ plus a peak velocity ≥4 m per second or a mean valve gradient ≥40 mm Hg) and high-risk status for procedural surgical aortic-valve replacement, as determined by experienced surgeons.. The subject or subject's legal representative has been informed of the nature of the study, agrees to its provisions and has provided written informed consent. Patients were considered to be at high procedural risk if they had coexisting conditions that were associated with a risk of death of at least 15% by 30 days after the operation.

### TAVR procedure

SAPIEN 3 aortic valve was used in accordance with the most current instruction for use. Valve Size was chosen according to heart team evaluation. TAVR procedure was almost according to the previous study (16). The valve implant procedure was considered to have started when the first interventional access-related puncture/incision was established. Size selection was made by CT image analysis based on the area of the autologous annulus, with adjustments made with reference to supravalvular structures such as sinuses of Valsava (SOV) and sinotubular junction (STJ). Preference for valve implantation was given to the right femoral artery approach, with replacement of the left femoral artery approach being performed if adverse features were present. Adverse features include: vascular access less than 5.5 mm with concomitant annular calcification, porcelain aorta, coronary opening less than 10 mm and small SOV diameter. Intraoperatively, if there was uncertainty about size selection on CT assessment, the presence of coronary risk or severe calcification that made it difficult to cross the valve, balloon pre-dilation was assessed first, otherwise the valve was implanted directly.

Physician monitors, trained clinical experts and medical affairs reviewed all data and confirmed the accuracy of data collection for analysis. Surgeons were involved in 292/438 procedures (66.7%). The data collection followed the Global THV case collection criteria. Patient characteristics, procedural characteristics and intraoperative clinical outcomes were primarily collected. The study was registered at the Chinese Clinical Trial Registry with the registration number: ChiCTR-ONC-17011730.

### Follow-up

The prespecified primary endpoint was all-cause mortality at 1 year for the pooled cohort. Prespecified secondary endpoints included cardiovascular mortality, stroke, repeat hospitalization, acute kidney injury, vascular complications, bleeding events, and New York Heart Association (NYHA) functional class. Follow-up assessments of clinical outcomes, transthoracic echocardiography, and electrocardiography were conducted via outpatient visits interviews at 1, 3, 6, and 12 months, and adverse events and prosthetic valve hemodynamics were recorded. All patients were followed for at least 1 year and had annual clinical visits and echocardiographic evaluations.

### Statistical analysis

Continuous variables were expressed as means ± standard deviations or medians with interquartile ranges as appropriate and were compared using *t*-test or nonparametric test as appropriate. Meanwhile, categorical variables were expressed as frequencies and percentages and were compared using the Chi-square test or the Fisher's exact test as appropriate. Graphpad 8.0 was used for statistical analysis. A two-sided *P* value of less than 0.05 was considered statistically significant.

## Results

All 438 cases since NMPA approval were collected from June 2020–May 2022. These cases were from 21 provinces and 74 implanted institutions. Among them, 197 female cases and 241 male cases, average age was 73.7 ± 8.6, average left ventricular ejection fraction was 57.2 ± 11.4. 223 patients with bicuspid aortic valve stenosis and 215 with tricuspid aortic valve stenosis were found. The average annulus size was 474.9 ± 97.5 mm^2^. In these cases, left coronary artery (LCA) height was 13.7 ± 3.2 mm, right coronary artery (RCA) height was 16.3 ± 3.0 mm. In these 438 cases, 6 cases had undergone Cardiac surgery, 77 cases had performed percutaneous coronary intervention. Detailed information of patient characteristics was involved in Table 1.

**Table 1:** Patient characteristics—overall population.

Parameters	mean ± SD or *n*/*N* (%)
Patient characteristics	(*n* = 438)
Age (years)	73.7 ± 8.6
Female(%)	197 (45.0)
Left ventricular ejection fraction (%)	57.2 ± 11.4
Valve anatomical conditions(%)	
Tricuspid Aortic Valve (TAV)	215/438 (49.1)
Bicuspid Aortic Valve (BAV)	223/438 (50.9)
Type 0	91/223 (40.8)
Type 1	127/223 (56.9)
Type 2	5/223 (2.2)
Annulus size (mm^2^)	474.9 ± 97.5
Coronary height (mm)	
Left coronary artery (LCA)	13.7 ± 3.2
Right coronary artery (RCA)	16.3 ± 3.0
LVEDD (cm)	5.63 ± 1.50
Peak aortic valve pressure(mmHg)	92.28 ± 26.05
Mean aortic pressure gradient(mmHg)	49.66 ± 8.81
Ascending Aorta diameter (mm)	37.42 ± 4.67
Annulus diameter (mm)	24.10 ± 2.51
LVOT diameter (mm)	24.87 ± 2.58
SoV diameter (mm)	28.43 ± 2.99
STJ diameter (mm)	26.51 ± 3.07
Operation history	
Cardiac surgical procedures	6/438 (1.4)
PCI	77/438 (17.6)

LVEDD, left ventricular end-diastolic dimension; LVOT, left ventricular outflow tract; SoV, sinus of Valsalva; STJ, sinotubular junction; PCI, percutaneous coronary intervention.

**Figure 1:**
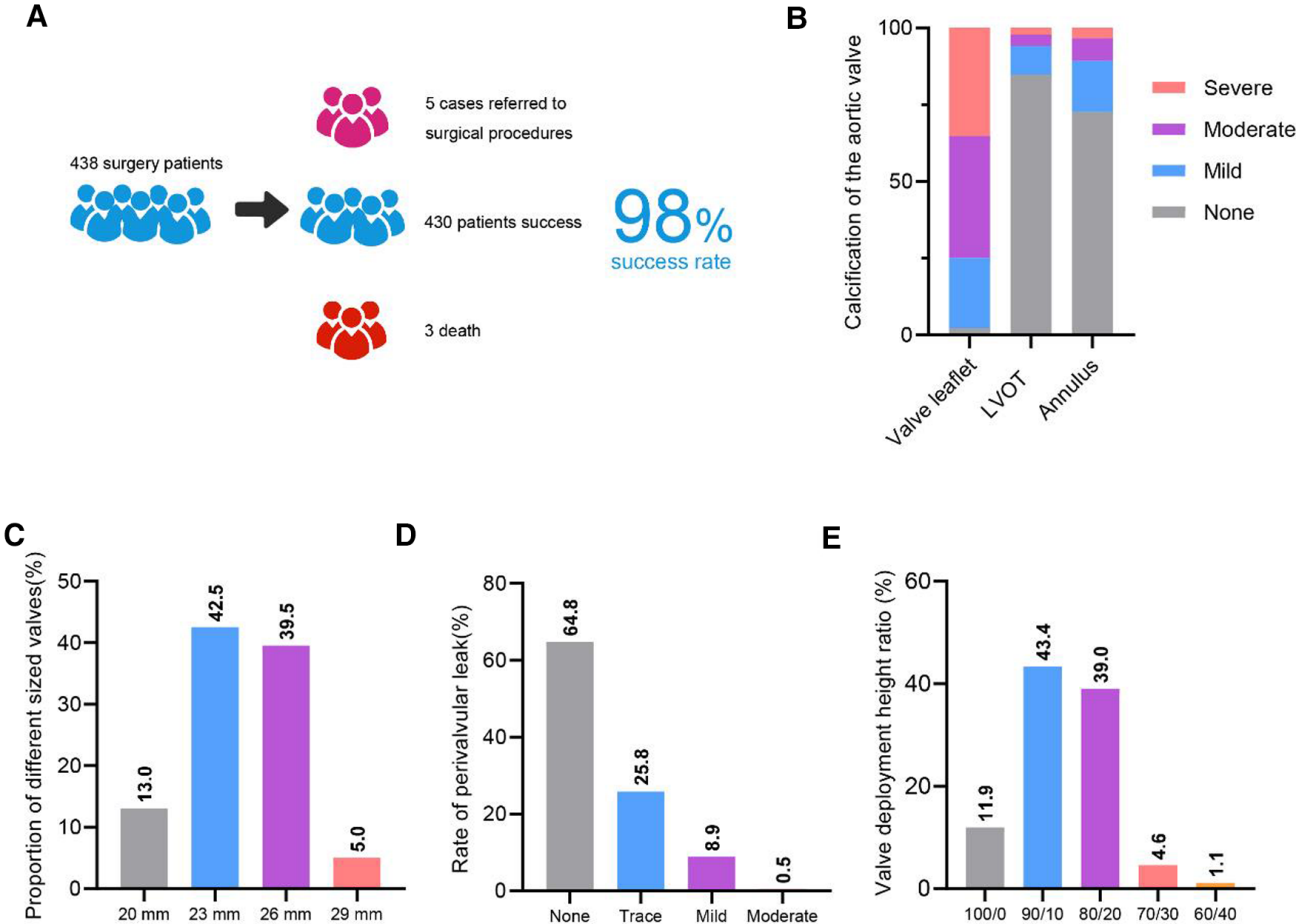
Overall clinical data. **A**. Diagram of the overall surgical success rate. **B**. Overall degree of calcification. LVOT, left ventricular outflow tract. **C**. Overall proportion of different sized valves. **D**. Overall different degrees of perivalvular leak. **E**. Overall valve deployment height.

For procedural outcomes, the procedure success rate was 98.2% (Figure 1A). 5 cases were converted to surgery during the procedure. Only 1 case implanted more than 1 valve. 12 cases were implanted with new permanent pacemaker and major vascular complication occurred in 1 patient. The 30-day mortality was 3.2% (Table 2).

**Table 2:** Procedural outcomes—overall population.

Parameters	Mean ± SD or *n*/*N* (%)
	(*n* = 438)
Procedural mortality	3/438 (0.7)
Procedure success rate^a^	430/438 (98.2)
Conversion to surgery^b^	5/438 (1.1)
Coronary obstruction during procedure	6/438 (1.4)
>1 valve implanted	1/438 (0.2)
New permanent pacemaker	12/438 (2.7)
Major vascular complication^c^	1/438 (0.2)
STS PROM %	8.47 ± 1.33
30-day mortality^d^	6/186 (3.2)

^a^ Procedure success defined as follows: 1 valve successfully implanted in the aortic valve position; no conversion to surgery, patient survived.

^b^ 1 surgery converted to elective surgery, 4 emergency surgeries.

^c^ According to the VARC-2 definition.

^d^ Full 30-day mortality information is not yet available.

STS PROM, Society of Thoracic Surgeons 30-day predicted risk of mortality.

We analyzed calcification of aortic valve in 438 cases, and found a high degree of moderate and severe leaflet calcification. Calcification in left ventricular outflow tract (LVOT) and annulus were low (Figure 1B). For valve size, the proportion of 23 mm and 26 mm valves was higher, at 42.5% and 39.5% respectively (Figure 1C). A low proportion of moderate perivalvular leak (0.5%) was found, no severe perivalvular leakage was found (Figure 1D). Valvular deployment height for 90/10 and 80/20 was 43.4% and 39%, respectively (Figure 1E).

In all patients, the follow-up period was 3 months–2 years, with a median follow-up time of 1.1 years. Outpatient or telephone follow-up and echocardiography were completed in about one year in 225 (51.4%) of all patients, showing a 1-year all-cause mortality rate of 6.2% (*n* = 14), including a cardiovascular mortality rate of 4.9% (*n* = 11), a cardiovascular-related readmission rate of 12.0% (*n* = 27) and a stroke rate of 3.6% (*n* = 8). Ultrasound follow-up results showed a 6.7% incidence of mild intervalvular or perivalvular regurgitation (*n* = 15), a 1.3% incidence of moderate or greater intervalvular or perivalvular regurgitation (*n* = 3), and a 4.4% incidence of moderate or greater stenosis (mean transvalvular pressure difference >20 mmHg) (*n* = 10).

We categorized 438 cases for BAV and tricuspid aortic valve (TAV). Among them, 223 cases were for BAV and 215 for TAV. For BAV cases, 102 females and 121 males, the average age was 71.6 ± 8.2. For TAV cases, 95 females and 120 males, the average age was 75.9 ± 8.4. No difference was found in gender and LVEF. The average annulus size in BAV was 492.5 ± 105.0 mm^2^, in TAV was 456.9 ± 85.8 mm^2^, size in BAV was significantly higher than TAV. Left coronary artery (LCA) height in BAV was 14.4 ± 3.3 mm, in TAV was 12.9 ± 2.9 mm. Right coronary artery (RCA) height in BAV was higher than in TAV (Table 3).

**Table 3:** Patient characteristics—BAV vs. TAV.

Parameter	BAV	TAV	*p*-value
	Mean ± SD or *n*/*N* (%)	Mean ± SD or *n*/*N* (%)	
Patient characteristics	(*n* = 223)	(*n* = 215)	
Age (years)	71.6 ± 8.2	75.9 ± 8.4	<0.001
Female (%)	102 (45.7)	95 (44.2)	0.744
LVEF (%)	57.7 ± 10.4 (*n* = 114)	56.7 ± 12.4 (*n* = 108)	0.486
Valve anatomical conditions			
LCA height (mm)	14.4 ± 3.3	12.9 ± 2.9	<0.001
RCA height (mm)	16.7 ± 2.9	16.0 ± 3.0	0.009

LCA, Left coronary artery; RCA, Right coronary artery; LVEF, Left ventricular ejection fraction.

We also analyzed procedural outcomes in BAV and TAV, the procedure success rate in BAV was 97.8%, in TAV was 98.6%. 4 cases in BAV and 1 case in TAV were converted to surgery during procedure. No difference was found in coronary obstruction during procedure, new permanent pacemaker and major vascular complication between BAV and TAV (Table 4).

**Table 4:** Procedural outcomes—bicuspid vs. Tricuspid Aortic Valves.

Parameter	BAV	TAV	*p*-value
	Mean ± SD or *n*/*N* (%) (*n* = 223)	Mean ± SD or *n*/*N* (%) (*n* = 215)	
Procedural mortality	1/223 (0.4)	2/215 (0.9)	0.617
Procedure success rate*	218/223 (97.8)	212/215 (98.6)	0.724
Conversion to surgery**	4/223 (1.8)	1/215 (0.5)	0.373
Coronary obstruction during procedure	1/223 (0.4)	5/215 (2.3)	0.116
>1 valve implanted	0/223 (0)	1/215 (0.5)	0.491
New permanent pacemaker	6/223 (2.7)	6/215 (2.8)	1.000
Major vascular complication***	1/223 (0.4)	0/215 (0)	1.000

*Procedure success is defined as follows: successful valve implantation in the aortic valve position without conversion to surgery and patient survival.

**1 operation converted to elective surgery and 4 emergency surgery.

***As defined by VARC-2.

Annulus size in BAV was 492.5 ± 105.0 mm, in TAV was 456.9 ± 85.8 mm, with a significant difference (*P* < 0.001). For valve sizing, oversized, within sized and undersized in BAV were 5 cases, 138 cases and 80 cases respectively, and in TAV were 8 cases, 186 cases and 21 cases respectively, with significant differences (Table 5).

**Table 5:** Valve sizing in BAV and TAV.

Parameter	BAV	TAV	*p*-value
	Mean ± SD or *n*/*N* (%)(*n* = 223)	Mean ± SD or *n*/*N* (%)(*n* = 215)	
Annulus size (mm^2^)	492.5 ± 105.0	456.9 ± 85.8	<0.001
Valve sizing^a^			<0.001
Oversized	5/223 (2.2)	8/215 (3.7)	
Within size	138/223 (61.9)	186/215 (86.5)	
Undersized	80/223 (35.9)	21/215 (9.8)	

^a^ Oversizing was defined as: measured annulus size < min. area declared by valve size. Undersized was defined as: measured annulus size > maximal area declared by valve size.

**Figure 2:**
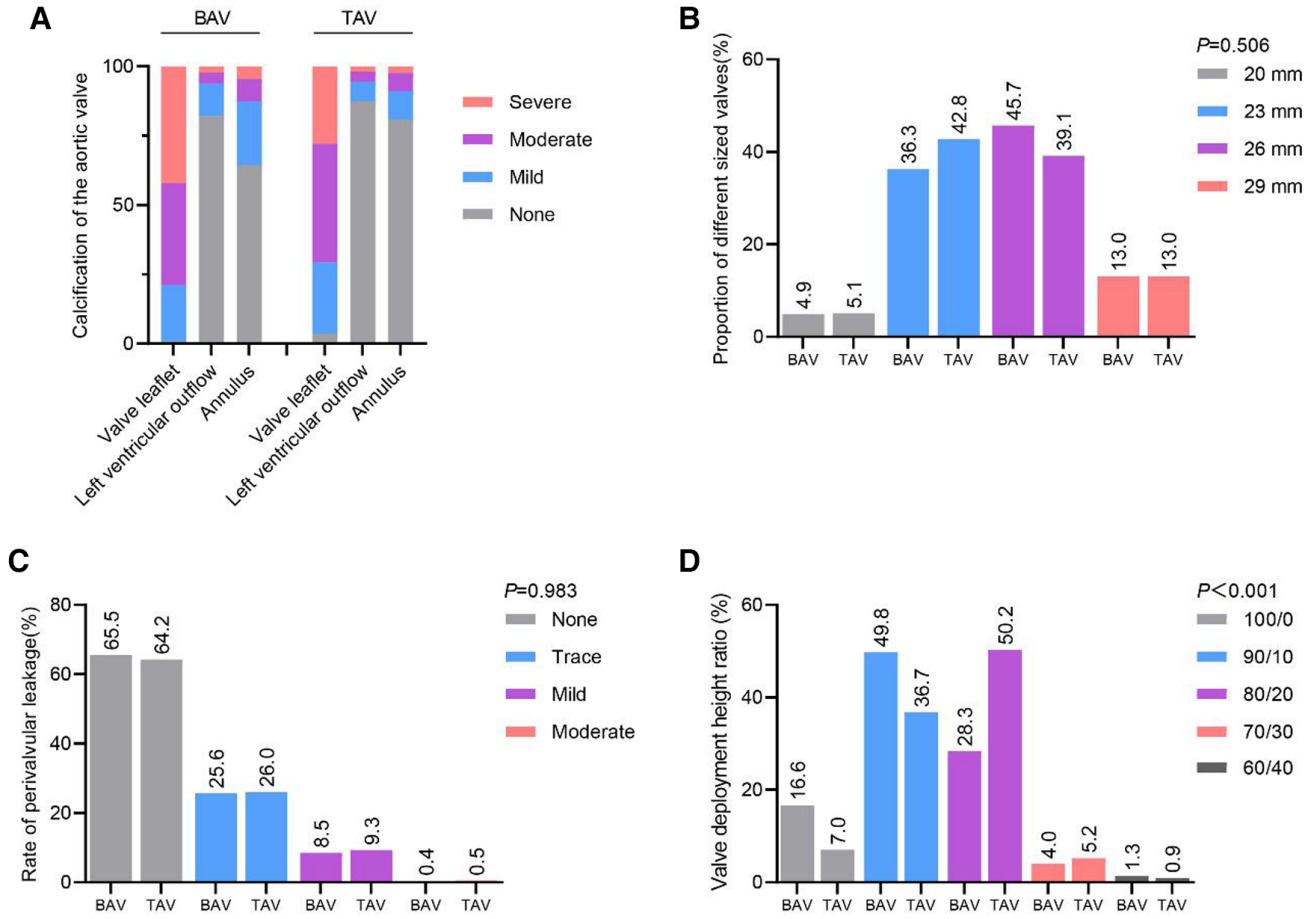
Clinical data in BAV and TAV. **A**. Degree of Calcification in BAV and TAV. **B**. No difference in implanted valve size between BAV and TAV. **C**. No difference in performance between BAV and TAV paravalvular leaks. **D**. Valve Deployment Height in BAV and TAV. BAV, Bicuspid aortic valves; TAV, Tricuspid aortic valves.

Leaflet calcification was high in both BAV and TAV. While calcification in left ventricular outflow tract (LVOT) and annulus were low in BAV and TAV (Figure 2A). No difference of valve size proportion was found between BAV and TAV (Figure 2B). For perivalvular leak, no significant difference was found between BAV and TAV (Figure 2C). Valvular deployment height in BAV was higher than TAV for 100/0 and 90/10 (Figure 2D).

## Comment

In Western countries, AS is a common heart condition in the elderly population, with the incidence increasing progressively with age. It is about 2% in people aged >65 years and about 4% in people aged >85 years. Exact epidemiological data on AS are not available in China. TAVR, also known as transcatheter aortic valve implantation (TAVI), is a functionally complete replacement of the aortic valve by placing a fully assembled prosthetic valve into the diseased aortic valve via a catheter (17, 18). Chinese TAVR candidates differ from Western countries in that a higher proportion of Chinese patients have BAV (19, 20). The proportion of patients with BAV in our study was 50.9%, which is generally consistent with previous results. The mean age of BAV is significantly younger than that of TAV in our study, this may be due to the earlier onset of symptoms associated with aortic stenosis in BAV patients, resulting in the younger average age of the BAV patients we observed. Similar results have been found in several studies (5, 15).

BAV is the most common congenital heart disease, with a prevalence of 1%–2% in the population (15). According to Sievers et al., BAV is classified as type 0, type 1 and type 2 according to the number of fused ridges (21). Previous studies have found that type 1 accounts for 70% of BAV in Western countries (22). In contrast, type 0 BAV is more common in Chinese patients (23). Our study found that type 1 BAV accounted for 57% and type 0 BAV accounted for 41%, with slightly inconsistent results, possibly due to our inadequate sample size or because we were analyzing BAV in AS patients instead of BAV in general.

In our study, a mortality rate of 3.2% was found at 30 days after procedure. A 30-day mortality rate of 5.4% was found in a US population SAPIEN 3 placement study (24). In a European multicenter study of 1,947 patients underwent TAVI with the SAPIEN 3, the 30-day mortality rate was found to be 2.2% (25). These findings were consistent with our results.

In TAVR, the appropriate valve is selected within the range of available sizes to ensure that the valve will best accommodate the native aortic annulus root (26). The aortic annulus usually represents the tightest part of the aortic root, and the size of the valve is traditionally dependent on the size of the systolic aortic annulus (27). Our study found that the size of the annulus was significantly larger in patients with BAV than in those with TAV, which also guides valve selection. Subsequently, more 26 mm valves were used in BAV patients, and smaller valves were used less frequently in BAV than in TAV. As the larger size 29 mm valve was used at a comparable rate in BAV vs. TAV, it was since the 29 mm valve was not available until March 2021, which affected the results.

In BAV, implantation of a 90/10 height is preferred. This strategy reduces the risk of valve migration into the ventricle, as well as the usually longer overhang of the native valve into the ventricle and permanent pacemaker implementation (PPI), in addition to the good results regarding perivalvular leak (28, 29). Olaf Wendler, et al. found a PPI of 12% in the European population after underwent TAVI with the SAPIEN 3, compared to 2.7% in our study (25). In our study, BAV deployment height of 90/10 accounted for nearly 50%, which is consistent with previous findings. TAV deployment heights of 80/20 accounted for 50.2% of the total, while 90/10 accounted for 36.7%. Deployment heights of BAV and TAV valves are significantly different. Hasan Jilaihawi et al. found that the degree of calcification in the leaflets was threefold higher in the Chinese population than in the Western population (30). In our study, the overall leaflet had a high degree of calcification and more moderate and severe calcification.

In first-generation valves, perivalvular leak is a common complication. Above moderate perivalvular leak can be up to 16.0% with self-expanding valves (CoreValve) and 9.1% with the ball-expanding valve SAPIEN (31). With the use of newer generation valves, the incidence of perivalvular leak is becoming less frequent. Many patients have a minor to mild perivalvular leak. Mohamed Abdel-Wahab (32) et al. found no statistically significant difference in the outcome of an early generation of BEV and SEV via transfemoral transcatheter aortic valve replacement. Makkar et al. also found comparable efficacy of BEV in the treatment of BAV vs. TAV (5). The SAPIEN 3 valve employs BEV, and in a retrospective study in BAV, BEV was found to have a lower incidence of perivalvular leak and PPI than SEV (33). Our study showed a moderate or greater perivalvular leak of 0.4% in BAV and 0.5% in TAV. The results for the incidence of perivalvular leak are relatively promising. In addition, there is no difference in the comparison of PPI in BAV as well as TAV.

Some limitations exist in our study. Firstly, as the SAPIEN 3 valve was only approved by the NMPA in June 2020 and information about the last cases was collected in May 2022, post-operative follow-up information was lacking, we need time to evaluate the follow-up data after valve implantation. In addition, with sufficient follow-up data, we would like to compare the clinical data of SAPIEN 3 valve in other Asian countries as well as in Western countries to more accurately assess the performance of SAPIEN 3 valve in Chinese patients.

Taken together, our study confirms that the SAPIEN 3 valve has displayed excellent clinical results in the treatment of Chinese patients with AS, achieving high procedural success rates in Chinese patients with complex valve morphology. Similar good clinical outcomes were achieved in patients with BAV and TAV, with only minimal perivalvular leak and low permanent pacemaker implantation in both valve types. The annulus size in BAV was significantly larger than TAV. The undersized valves in BAV group were significantly more than TAV group. Coronaries height in both left and right were in BAV group than BAV. Moreover, valve deployment height differs between BAV and TAV. Therefore, achieving a good BAV or TAV effect requires an appropriate valve size and deployment height.

## Data Availability

The original contributions presented in the study are included in the article, further inquiries can be directed to the corresponding author/s.
